# Examining the Relationship Between Academic Excellence and Clinical Productivity in Orthopedic Surgery

**DOI:** 10.3390/jcm15051900

**Published:** 2026-03-02

**Authors:** Mohamad Y. Fares, Xiaoran Zhang, Harry H. Liu, Ana Paula Beck da Silva Etges, Krishna Chopra, Brian Zhou, Ira Sivaram, Chrishaun Alexander, Peter Boufadel, Porter Jones, Derek A. Haas, Adam Z. Khan, Eric Wagner, Joseph A. Abboud

**Affiliations:** 1 Rothman Institute, Thomas Jefferson University Hospital, Philadelphia, PA 19107, USA; 2 Department of Orthopaedic Surgery, University of Pittsburgh Medical Center, Pittsburgh, PA 15213, USA; 3 Avant-garde Health, Boston, MA 02111, USAderek@avantgardehealth.com (D.A.H.); 4 Department of Medicine, Baylor Scott & White Health, Temple, TX 76508, USA; 5 Department of Orthopaedic Surgery, Emory School of Medicine, Atlanta, GA 30322, USAbrian.zhou@emory.edu (B.Z.); irasivaram8@gmail.com (I.S.);; 6 Southern California Permanente Medical Group, Department of Orthopaedic Surgery, Panorama City, CA 92887, USA

**Keywords:** publication, clinical volume, RVU, Healthgrades, all-stars

## Abstract

**Background/Objectives:** The relationship between clinical volume and academic performance in orthopedic surgery remains understudied. The purpose of this study is to explore the characteristics of high-achieving academic orthopedic surgeons in an attempt to extrapolate patterns and trends that govern the relationship between clinical performance and academia in orthopedic surgery. **Methods:** The 2023 National Plan and Provider Enumeration System and Medicare claims data (2021–2022) databases were used to include all active orthopedic surgeons of different subspecialties. A publication score, based on publication volume, journal impact, and authorship position, was calculated for each included surgeon, and surgeons who scored in the top 5% were deemed high-achieving academic orthopedic surgeons. Additional data pertaining to demographic characteristics, clinical volume, relative value units (RVUs), and Healthgrades ratings were recorded and analyzed. **Results:** A total of 23,403 orthopedic surgeons were included in our study, with 1169 considered top researchers. There were significant disparities in multiple parameters according to gender. Moreover, there were geographic variations among orthopedic surgeons with regard to mean publication scores, clinical volume, and RVUs. The top researcher cohort had a higher mean publication score (*p* < 0.001) and a higher mean clinical volume (*p* < 0.001) when compared to the total surgeon cohort. Mean RVUs were higher in the total surgeon cohort, although not reaching significance. Hip and knee, as well as shoulder and elbow surgeons, had significantly greater clinical volumes in the top researcher cohort than in the total surgeon cohort (*p* < 0.001). Despite differences in clinical and research metrics, there were no significant differences in mean Healthgrades ratings and the mean number of Healthgrades ratings between the top researcher sample and the non-top researcher sample. **Conclusions:** Higher research productivity was not associated with lower clinical productivity, as high-achieving academic orthopedic surgeons demonstrated high academic performance while remaining clinically active.

## 1. Introduction

In the setting of orthopedic surgery, clinical productivity and research can have a symbiotic relationship [1,2,3]. Research helps advance surgical techniques, improve patient outcomes, and inform the best surgical practices in the field, while clinical productivity exposes surgeons to diverse cases, leading to increased research opportunities and the provision of valuable data [2]. However, involvement in research can detract from clinical practice, potentially reducing clinical volume and experience [2]. As such, there exists a notion that surgeons who are heavily involved in research may not be as skilled or dedicated in practice compared to those who are not [4].

Given the complex nature of the relationship between research productivity and clinical practice in orthopedic surgery, understanding demographic and clinical trends among high-achieving academic orthopedic surgeons can provide insight into the characteristics associated with high academic performance and areas of potential improvement in the field.

To date, limited work has examined the epidemiological characteristics of academic orthopedic surgeons, and few studies explored the relationship between clinical performance and research productivity in orthopedic surgery. Prior studies suggest that physician workforce characteristics, academic engagement, and practice setting are associated with varying patterns of clinical activity and healthcare delivery [5,6,7]. These frameworks offer a useful lens for assessing the contribution of orthopedic surgeons to health systems through clinical and academic activity, while taking into account patient experience. Accordingly, the aim of our study is to explore the demographic, geographic, academic, and clinical characteristics of top orthopedic surgery researchers through different variables and metrics, while drawing comparisons to the total cohort of orthopedic surgeons.

## 2. Materials and Methods

### 2.1. Data Collection and Surgeon Inclusion

Surgeons were identified using the 2023 National Plan and Provider Enumeration System (NPPES) file published by the Centers for Medicare and Medicaid Services (CMS) [8]. Various data were collected for all orthopedic surgeons, including surgeon gender, geographic location, medical school graduation year, number of publications, publication score, number of clinical procedures, number of relative value units (RVU), and orthopedic subspecialty. Subspecialties included were hip and knee, foot and ankle, shoulder and elbow, hand and wrist, generalists, and spine surgery. Surgeons were required to report their orthopedic subspecialty in order to be included in our study. Additionally, surgeons in training who graduated from medical school within the past six years were excluded.

To ensure accurate specialty attribution, surgeons were further screened based on their Medicare procedure volume and recent graduation status. Procedure volume data were obtained from 2021 to 2022 Medicare fee-for-service (FFS) inpatient and outpatient claims, identified using Diagnosis-Related Groups (DRGs) and Current Procedural Terminology (CPT) codes [9]. Surgeons performing fewer than ten procedures during this period were excluded from the analysis. Surgical specialty classification was determined based on Medicare procedural volume, with surgeons classified into a specific specialty if at least 50% of their procedures were within that domain.

Peer-reviewed articles published from 2021 to 2023 were retrieved from the PubMed database. This extended publication window was selected to account for delays between study completion, journal acceptance, and indexing in PubMed, thereby ensuring more complete capture of recent and relevant publications. Surgeon names were matched to article authors using a combination of name matching and affiliation matching. Letters to the editor were excluded from the analysis.

### 2.2. Calculating Publication Score

Each publication was initially assigned a base score of one point, which was subsequently adjusted based on two factors: journal impact factor and author position. The weighting scheme for author positions was as follows:

- Single author: 100% of the score.
- Two authors: First author = 60%, last author = 40%.
- Three or more authors: First author = 45%, last author = 30%, and the remaining authors shared 25%, divided equally among them.
- Author-specific weighted individual article score = Journal Impact Factor × Author Position Weight.

The weighted publication score for each surgeon was calculated by summing the author-specific weighted individual article scores across all attributed articles. Surgeons were then ranked within their respective specialties based on their total weighted publication scores. The top 5% of orthopedic surgeons were designated as orthopedic surgery “Top Researchers.”

### 2.3. Healthgrades Analysis

A subsequent Healthgrades (Denver, CO, USA) analysis was conducted to assess and evaluate patient satisfaction and grading between surgeons who were in the top researcher cohort and those who were not [10]. A 5% random sample was generated from each group (top researchers vs. non-top researchers), while standardizing according to physician subspecialty, gender, and geographic location. Both Healthgrades ratings and the number of Healthgrades ratings were recorded for the 5% representative sample of each group.

### 2.4. Statistical Analysis

A descriptive statistical analysis was conducted in order to appropriately represent the data in our study. An independent *t*-test was conducted to compare different demographic, clinical, and academic variables between the top researcher cohort and the total surgeon cohort. An independent *t*-test was also used to compare the mean Healthgrades ratings and the mean number of Healthgrades ratings between the two cohorts. An analysis of variance (ANOVA) test was conducted to compare the mean Healthgrades ratings and the mean number of Healthgrades ratings between different orthopedic subspecialties. A Games–Howell or a Tukey’s test was used as a post hoc test depending on whether the assumption of homogeneity of variances was violated or not. A *p*-value of less than or equal to 0.05 was considered significant. Statistical analyses were performed using IBM SPSS Statistics for Windows, Version 26.0 (IBM Corp., Armonk, NY, USA).

## 3. Results

### 3.1. Distribution by Gender

A total of 23,403 orthopedic surgeons were included in our study. These surgeons were distributed according to the following specialties: 10,450 hip and knee surgeons (44.7%), 5268 spine surgeons (22.5%), 3437 general orthopedists (14.7%), 1879 hand surgeons (8.0%), 1528 shoulder and elbow surgeons (6.5%), and 841 foot and ankle surgeons (3.6%). The orthopedic top researcher cohort, i.e., top 5% of surgeons with regard to research productivity, constituted 1169 of the total cohort, distributed across different orthopedic subspecialties: 462 hip and knee surgeons (39.5%), 263 spine surgeons (22.5%), 181 general orthopedists (15.5%), 150 shoulder and elbow surgeons (12.8%), 55 hand surgeons (4.7%), and 58 foot and ankle surgeons (5.0%). Females constituted 6.8% (N = 1594) of our total surgeon cohort and 7.1% (n = 83) of the top researcher cohort; there was no significant difference with regard to representation between the total surgeon cohort and the top researcher cohort (*p* = 0.503). Table 1 and Table 2 show the breakdown of the included orthopedic surgeons in each cohort by gender and orthopedic subspecialty.

When conducting statistical analysis, it was seen that male orthopedic surgeons have a significantly higher mean clinical volume (131.6 vs. 95 procedures, *p* < 0.001), a higher mean publication score (6.3 vs. 4.3, *p* = 0.001), and a higher mean number of RVUs (268 vs. 161.1, *p* < 0.001). It was also seen that the average male orthopedic surgeon included in our study graduated from medical school significantly earlier than the average female orthopedic surgeon (1999 vs. 2005, *p* < 0.001).

### 3.2. Distribution of Average Clinical Volume, RVUs, and Publication Score by State

The states of California, Texas, and Florida had the highest number of included surgeons, with 1868 (8.09%), 1674 (7.25%), and 1432 (6.2%) orthopedic surgeons, respectively. On the other hand, the states of New York, California, and Pennsylvania had the highest rate of top researchers, with 166 (14.22%), 121 (10.37%), and 77 (6.6%) orthopedic top researchers, respectively.

When comparing different states according to different productivity parameters, South Dakota, North Dakota, and Kansas had the highest average clinical volumes with 270.82, 242.26, and 214.66 procedures per orthopedic surgeon. Delaware, Washington, D.C., and South Carolina had the highest average RVUs, with 483.46, 370.76, and 353.6 RVUs per orthopedic surgeon. As for average publication score, Illinois, Minnesota and Massachusetts had the highest average publication score, with scores of 4.38, 4.36, and 3.95 points per orthopedic surgeon, respectively.

### 3.3. Distribution by Graduation Year

**Figure 1:**
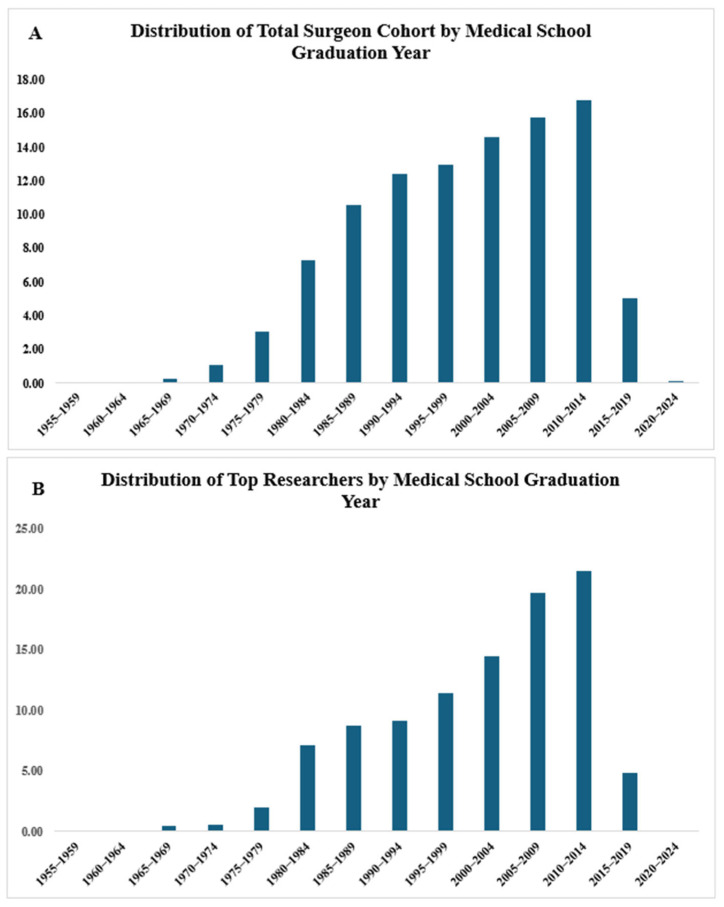
Distribution of the total surgeon cohort (**A**) and the top researcher cohort (**B**) by their medical school graduation year.

The distribution of the total surgeon cohort and the top researcher cohort by medical school graduation year was assessed. Figure 1A shows the distribution of the total surgeon cohort by their medical school graduation year. The timeframes of 2005–2009 and 2010–2014 saw the graduation of the highest percentages of surgeons, at 15.77% and 16.76% respectively. Figure 1B shows the distribution of the top researcher cohort by their medical school graduation year. Similarly, 2005–2009 and 2010–2014 saw the graduation of the highest percentages of top researchers. However, percentages were higher in the top researcher cohort, at 19.68% and 21.48% during these two time periods, respectively. Overall, both cohorts demonstrated a similar distribution with regard to medical school graduation years.

### 3.4. Comparing Clinical Volume, RVUs, and Publication Score

Our total surgeon cohort contributed 3,021,140 clinical procedures, 6,011,840 RVUs, 35,492 publications, and 13,019 publication score points. When comparing the total orthopedic surgeon cohort and the top researcher cohort, the average clinical volume was significantly higher in the top researcher cohort, with 147 procedures, when compared to the total surgeon cohort with 129 procedures (*p* < 0.001). However, average RVUs were higher in the total surgeon cohort with 256.88 RVUs when compared to the top researcher cohort with 246.58 RVUs, although this difference did not reach statistical significance (*p* = 0.117). With regard to research productivity, the average publication volume and average publication scores were 1.52 and 0.56 points for the total surgeon cohort, respectively, which were significantly lower than those of the top researcher cohort with 20.05 publications (*p* < 0.001) and 9.27 points (*p* < 0.001), respectively. Table 3 and Table 4 show the total and average clinical volume, publication volume, publication score, and RVUs for both the total surgeon cohort and the top researcher cohort.

When comparing the clinical productivity of different orthopedic subspecialties between the total surgeon cohort and the top researcher cohort, it was found that hip and knee surgeons and shoulder and elbow surgeons had a significantly higher mean clinical volume in the top researcher cohort when compared to the total surgeon cohort (*p* < 0.001 each). Other subspecialties did not demonstrate differences in clinical volume between the total surgeon cohort and the top researcher cohort. However, when comparing the RVUs of different orthopedic subspecialties between the two cohorts, it was found that general orthopedists in the total surgeon cohort demonstrated significantly higher mean RVUs than those in the top researcher cohort (*p* < 0.001), with no other differences among subspecialties of the two cohorts. Comparisons of clinical volume and RVUs of different orthopedic subspecialties from each cohort can be seen in Table 5 and Table 6.

### 3.5. Comparing Healthgrades Scores

A 5% sample of our total surgeon cohort was calculated and randomly selected to conduct a Healthgrades ratings analysis in order to determine any correlations or associations between surgeon ratings and top researcher status. Samples were calculated while taking into account top researcher status and surgeon specialty. The top researcher sample consisted of 59 surgeons, and the non-top researcher sample consisted of 1111 surgeons from different orthopedic subspecialties. Five surgeons from the top researcher cohort and 46 surgeons from the non-top researcher cohort did not have Healthgrades information and were thus excluded from the analysis. Table 7 and Table 8 shows the distribution of the surgeon samples included in our study.

Among the top researcher sample, hand and wrist surgeons showed the highest average Healthgrades ratings at 4.8/5, whereas shoulder and elbow surgeons received the highest mean number of Healthgrades ratings, with an average of 59 ratings. Among the non-top researcher sample, shoulder and elbow surgeons had the highest mean Healthgrades ratings at 4.5/5, as well as the highest number of Healthgrades ratings, with an average of 64 ratings. Table 7 and Table 8 show the mean Healthgrades ratings and the mean number of Healthgrades ratings of surgeons from different subspecialties in both samples.

The mean Healthgrades ratings were very similar between the top researcher sample (4.26/5) and the non-top researcher sample (4.31/5), entailing no significant differences between the scores of these two samples (*p* = 0.828). With regard to the mean number of Healthgrades ratings, top researcher surgeons received 37 ratings, whereas the non-top researcher surgeons received 45.8 ratings. That being said, no significant differences were seen with statistical analysis (*p* = 0.294).

Mean Healthgrades ratings and the mean number of Healthgrades ratings were also compared between different subspecialties in our cohort. Among the non-top researcher cohort, shoulder and elbow surgeons had a significantly higher mean Healthgrades rating when compared to spine surgeons (*p* = 0.003). No other statistically significant differences were found when comparing mean Healthgrades ratings between different subspecialties in our cohort. Generalist orthopedic surgeons received a significantly lower mean number of Healthgrades ratings when compared to hip and knee surgeons (*p* = 0.05), shoulder and elbow surgeons (*p* = 0.01), and spine surgeons (*p* = 0.015). Among the top researcher cohort, there were no significant differences between the mean Healthgrades ratings (*p* = 0.763) nor the mean number of Healthgrades ratings received (*p* = 0.531) for the different subspecialties.

## 4. Discussion

Our study showed that top orthopedic researchers showed similar gender distributions, medical school graduation year distributions, higher research productivity, and higher clinical volume but similar RVUs when compared to the total orthopedic surgeon cohort. Illinois, Massachusetts, and Minnesota were the states with the highest average publication scores, while South Dakota, North Dakota, and Kansas had the highest average clinical volume. Additionally, Delaware, Washington, D.C., and South Carolina had the highest average RVUs out of all other states.

Despite efforts to improve gender diversity in orthopedic surgery [11,12,13,14], the representation of female orthopedic surgeons remains low among both the total surgeon cohort (6.8%) and the top researcher cohort (7.1%), indicating a need for additional initiatives to mitigate this gender disparity. Male surgeons also had higher mean clinical volume, publication scores, and numbers of RVUs, raising questions about the systemic differences in opportunities and support present for male and female orthopedic surgeons. Several studies have shown that females in surgical specialties often face barriers such as fewer research opportunities, limited mentorship, and implicit bias, which may explain the findings in our study [15,16]. Another interesting finding was that the average female surgeon in our study graduated from medical school six years later than the average male surgeon, highlighting the more recent, though steadily increasing, integration of women into a field historically dominated by men.

While different subspecialties showed similar proportions between the total surgeon cohort and the top researcher cohort, shoulder and elbow surgery showed a notably higher proportion of top researchers, whereas hand surgery had a notably lower proportion of top researchers when compared to their distribution in the total surgeon cohort. This may reflect the different research interests and productivity between different orthopedic subspecialties [17].

Notable patterns were revealed when examining the geographic distribution of the surgeons in our study. The largest numbers of orthopedic surgeons were found in California, Texas, and Florida, which makes sense, given these are the most populated states in the United States [18]. That being said, the largest number of orthopedic top researchers was found in New York, California, and Pennsylvania, potentially due to a higher concentration of academic institutions, reflecting a higher interest in research and academia in these states [19]. Rural states like South Dakota and North Dakota showed higher clinical volumes, likely reflecting the different healthcare delivery challenges in these areas [20]. Less populated areas tend to have fewer surgeons, and this may lead to a broader coverage of orthopedic services, leading to higher clinical volumes [20]. This can also reflect a higher frequency of Medicare cases in these states. When looking at publication scores, Illinois, Minnesota, and Massachusetts were the leading states, indicating higher research productivity in these states and a higher concentration of productive academic surgeons. These geographic patterns suggest a dichotomy where urban areas seem to focus more on research productivity and rural areas seem to focus more on clinical care delivery.

A consistent trend was seen when exploring the medical school graduation years of orthopedic surgeons in our study. The majority of orthopedic surgeons in the two cohorts graduated between 2005 and 2014. However, this was more pronounced in the orthopedic top researcher cohort, which showed a higher percentage of surgeons graduating during that period. This suggests that, compared to older orthopedic surgeons, younger orthopedic surgeons possess a higher focus on research productivity and increasingly attempt to balance research endeavors with clinical practice [21].

Our study also showed that the top researcher cohort demonstrated significantly higher clinical volume compared with the overall orthopedic surgeon cohort, underscoring the ability of high-volume surgeons to excel in both academic and clinical domains. Although the slightly higher RVUs observed in the total surgeon cohort were not statistically significant, this difference may reflect variations in practice environment and case mix, where surgeons in certain geographic or institutional settings perform higher-complexity or revenue-generating procedures that yield comparable RVUs, despite differences in procedural volume.

Among subspecialties, it was seen that shoulder and elbow surgeons and hip and knee surgeons in the top researcher cohort had higher clinical volumes when compared to their counterparts in the total surgeon cohort. This finding shows that professional differences in subspecialties exist, and some may provide greater opportunities for integrating research pursuits with clinical endeavors. In this same setting, general orthopedists from the total surgeon cohort showed higher RVUs than compared to their counterparts in the top researcher cohort, reflecting differences in billing patterns or case complexities between subspecialists and generalists.

We did not find any significant differences between the mean Healthgrades ratings and the mean number of Healthgrades ratings between surgeons from the top researcher and the non-top researcher cohorts. This finding shows that patient satisfaction is not correlated with the academic performance and research productivity of orthopedic surgeons. It is possible that other factors like communication skills, provision of adequate education, and clinical outcomes play a greater role in shaping the patient experience within the clinical orthopedic experience [7,22,23]. Moreover, shoulder and elbow surgeons among the non-top researcher cohort showed higher mean Healthgrades ratings when compared to spine surgeons, indicating differences in patient experiences across the different subspecialties [24,25]. General orthopedists also received lower numbers of Healthgrades ratings than other specialties, potentially entailing lower levels of patient engagement or fewer interactions in broader clinical practices.

One caveat to studying the relationship between clinical productivity and research involves the individualized work structure of each surgeon. With the current available data, it is hard to capture whether scholarly work was conducted during protected research time, with institutional academic support, or outside scheduled clinical hours. The heterogeneity in practice environments constitutes a significant hurdle in comprehensively exploring this relationship. Nevertheless, it is plausible that some surgeons conduct research work during their time off, but this raises important considerations regarding workload and burnout.

Moreover, our study sheds light on the need for interventions that endorse equity, innovation, and productivity in orthopedic surgery. Supporting rural surgeons with academic opportunities, fostering collaborations between clinical and academic surgeons, providing strategies that balance clinical workload with research responsibilities, and addressing gender disparities among subspecialties are all recommendations that can help enhance the overall impact and vision of orthopedic surgery in the United States. In this setting, future research should explore different factors and variables that underlie the trends exhibited in our study, like faculty effort allocation, burnout, equity, and geographic limitations, and perhaps compare the trends seen in orthopedic surgery to those in other medical specialties.

Our study is the first to examine the complex relationship and patterns between academic involvement and clinical productivity among orthopedic surgeons. However, several limitations should be acknowledged. Our analysis was limited to surgeons listed in the CMS database, which excludes many highly productive academic orthopedic surgeons. Our analysis also included procedures performed at hospital-based centers, without taking into account any procedures performed at ambulatory surgical centers. In addition, geographic comparisons were subject to unmeasured confounding related to population density, institutional and academic center distribution, regional practice patterns, and variability in billing practices, which are not fully captured in administrative datasets. Furthermore, because women comprised a small proportion of the cohort, gender-stratified comparisons may be underpowered and should be interpreted descriptively; additionally, productivity measures were restricted to recent years to reduce confounding by career length. Additionally, other important measures of productivity, such as administrative responsibilities and teaching hours, were not included, as they are not captured by clinical metrics. Moreover, we excluded orthopedic trauma from our study, as it was hard to identify orthopedic trauma cases from the databases at hand, and we did not want to use inaccurate data in our analysis. Despite these limitations, our study provides valuable insights into the contrasting relationship between academic engagement and clinical productivity in orthopedic surgery. These findings help identify areas for improvement and inform potential recommendations.

## 5. Conclusions

Our study highlighted key patterns and trends among orthopedic surgeons, including gender disparities, professional differences according to geographic locations, and the balance between clinical productivity and research performance. Even though significant efforts are employed to improve female representation in orthopedic surgery, gender disparities remain notable in the field. In addition, geographic analysis showed that urban areas were more likely to be focused on research than rural regions, potentially due to different healthcare demands and deliveries. Notably, top researchers demonstrated higher clinical procedure volumes while maintaining similar RVUs to the total surgeon cohort, challenging the notion that being involved in research takes away from clinical practice. Moreover, there were no significant differences in patient satisfaction between surgeons who were top researchers and those who were not, suggesting that research productivity may not be a primary determinant of patient experience.

The findings of this study highlight areas for further investigation, including factors associated with gender disparities, geographic variation in research activity, and the relationship between clinical workload and research productivity among orthopedic surgeons.

## Figures and Tables

**Table 1:** Distribution of the total orthopedic surgeon cohort by gender.

Subspecialty	Females, N (%)	Males, N (%)	Total, N (%)
Foot & ankle	110 (13.1)	731 (86.9)	841 (3.6)
General orthopedics	353 (10.3)	3084 (89.7)	3437 (14.7)
Hand & wrist	300 (16.0)	1579 (84.0)	1879 (8.0)
Hip & knee	475 (4.6)	9975 (95.4)	10,450 (44.7)
Shoulder & elbow	96 (6.3)	1432 (93.7)	1528 (6.5)
Spine	260 (4.9)	5008 (95.1)	5268 (22.5)
Total	1594 (6.8)	21,809 (93.2)	23,403 (100)

**Table 2:** Distribution of the top orthopedic surgery researchers cohort by gender.

Subspecialty	Females, N (%)	Males, N (%)	Total, N (%)
Foot & ankle	5 (8.7)	53 (91.4)	58 (5.0)
General orthopedics	19 (10.5)	162 (89.5)	181 (15.5)
Hand & wrist	5 (9.1)	50 (90.9)	55 (4.7)
Hip & knee	31 (6.71)	431 (93.3)	462 (39.5)
Shoulder & elbow	9 (6.0)	141 (94.0)	150 (12.8)
Spine	14 (5.32)	249 (94.7)	263 (22.5)
Overall	83 (7.1)	1086 (92.9)	1169 (100)

**Table 3:** Distribution of the total and average clinical volume, publication volume, publication score, and RVUs for the total surgeon cohort.

Specialty	Clinical Volume	Avg. Clinical Volume	Publication Volume	Avg. Publication Volume	Total Publication Score	Avg. Publication Score	Total RVU	Average RVUs	Total Number of Cohort
**Foot & Ankle**	80,487	95.70	1473.00	1.75	494.1	0.59	160,758.59	191.15	841.00
**Generalist**	445,942	129.75	4666.00	1.36	1881.1	0.55	617,938.37	179.79	3437.00
**Hand & Wrist**	210,914	112.25	1928.00	1.03	655.4	0.35	359,806.37	191.49	1879.00
**Hip & Knee**	1,545,184	147.86	11,961.00	1.14	5058.4	0.48	2,776,550.50	265.70	10,450.00
**Shoulder & Elbow**	156,840	102.64	4038.00	2.64	1594.9	1.04	411,539.14	269.33	1528.00
**Spine**	581,773	110.44	11,426.00	2.17	3335	0.63	1,685,247.85	319.90	5268.00
**Total**	3,021,140	129.09	35,492.00	1.52	13,019	0.56	6,011,840.82	256.88	23,403.00

**Table 4:** Distribution of the total and average clinical volume, publication volume, publication score, and RVUs for the top orthopedic researchers cohort.

Specialty	Clinical Volume	Avg. Clinical Volume	Publication Volume	Avg. Publication Volume	Total Publication Score	Avg. Publication Score	Total RVU	Average RVUs	Total Number of Cohort
**Foot & Ankle**	6099.00	105.16	819.00	14.12	386.8	6.67	9058.38	156.18	58.00
**Generalist**	24,303.00	134.27	2917.00	16.12	1588.1	8.77	23,734.00	131.13	181.00
**Hand & Wrist**	6975.00	126.82	1114.00	20.25	512.9	9.32	9210.24	167.46	55.00
**Hip & Knee**	85,547.00	185.17	8258.00	17.87	4347.5	9.41	117,705.88	254.77	462.00
**Shoulder & Elbow**	19,934.00	132.89	3154.00	21.03	1429.1	9.53	39,231.21	261.54	150.00
**Spine**	29,470.00	112.05	7174.00	27.28	2573.3	9.78	89,316.78	339.61	263.00
**Total**	172,328	147	23,436	20.05	10,838	9.27	288,256.49	246.58	1169

**Table 5:** Comparisons of clinical volume between the total cohort and the top researcher cohort by subspecialty.

	Mean Clinical Volume—Total Cohort	Mean Clinical Volume—Top Researcher Cohort	t-Stat	*p*-Value
**Generalist**	129.75	134.27	−0.41	0.68
**Hip & Knee**	**147.86**	**185.17**	**−5.76**	**<0.001**
**Shoulder & Elbow**	**102.64**	**132.89**	**−4.16**	**<0.001**
**Foot & Ankle**	95.70	105.16	−0.92	0.36
**Hand & Wrist**	112.25	126.82	−1.01	0.31
**Spine**	110.44	112.05	−0.28	0.78
**Total**	**129.09**	**147.41**	**−4.96**	**<0.001**

**Table 6:** Comparisons of RVUs between the total cohort and the top researcher cohort by subspecialty.

	Mean RVU—Total Cohort	Mean RVU—Top Researcher Cohort	t-Stat	*p*-Value
**Generalist**	**179.79**	**131.13**	**3.97**	**<0.001**
**Hip & Knee**	265.70	254.77	0.87	0.38
**Shoulder & Elbow**	269.33	261.54	0.24	0.81
**Foot & Ankle**	191.15	156.18	1.58	0.11
**Hand & Wrist**	191.49	167.46	1.32	0.19
**Spine**	319.90	339.61	−1.53	0.13
**Total**	256.88	246.58	1.57	0.117

**Table 7:** Average Healthgrades ratings and average number of Healthgrades ratings among the representative sample of the top researcher cohort.

Subspecialty	N	Average Healthgrades Ratings	Average Number of Healthgrades Ratings
**Foot and Ankle**	3	4.6	18.8
**Hand and Wrist**	3	4.8	33
**Hip and Knee**	23	4.3	41.6
**Shoulder and Elbow**	8	4.4	59
**Spine**	13	4	34.3
**General Orthopedics**	9	4.35	17.9
**Total**	59	4.3	37

**Table 8:** Average Healthgrades ratings and average number of Healthgrades ratings among the representative sample of the non-top researcher cohort.

Subspecialty	N	Average Healthgrades Ratings	Average Number of Healthgrades Ratings
**Foot and Ankle**	39	3.95	38
**Hand and Wrist**	91	4.39	31.2
**Hip and Knee**	499	4.28	44
**Shoulder and Elbow**	69	4.5	64
**Spine**	250	4.2	48.7
**General Orthopedics**	163	4.4	31.1
**Total**	1111	4.3	45.8

## Data Availability

The data presented in this study are available upon request from the corresponding author (data are not publicly available due to privacy or ethical restrictions).

## Abbreviations

The following abbreviations are used in this manuscript:

MDPI	Multidisciplinary Digital Publishing Institute;
DOAJ	Directory of open access journals’
TLA	Three-letter acronym;
LD	Linear dichroism.
